# Modified triple pelvic osteotomy for residual acetabular dysplasia through double incisions: Technical note and review of short-term results

**DOI:** 10.1051/sicotj/2024012

**Published:** 2024-04-30

**Authors:** Ahmad S. Aly, Tamer A. Fayyad, Shady S. El-beshry, Karim T. Elhusseiny, Ahmed K. El Ghazawy

**Affiliations:** Department of Orthopedics surgery, Faculty of Medicine Ain Shams University Abbasia square Nasr City Cairo Egypt

**Keywords:** Acetabular dysplasia, DDH, Pelvic osteotomy, Triple pelvic osteotomy, PAO

## Abstract

*Purpose*: To assess validity, safety, and efficacy of the modified triple pelvic osteotomy (TPO) approach for correction of residual acetabular dysplasia. *Methods*: This is a retrospective case series conducted on 15 hips in 15 patients from 2019 to 2023 with residual acetabular dysplasia treated by modified TPO as described by Tonnis with two modifications. The first modification is using a single medial incision for pubic and ischial cuts (the Vladimirov modification). The second modification is having the ischial cut closer to the acetabulum (Li modification) allowing free movement of the acetabular fragment for better femoral head coverage. The mean age at the time of surgery was 11.85 years, (range 8–23). Cases presenting were 10 males (66.7%) and 5 females (33.3%). The mean follow-up period was 36.533 months (24–60 months). *Results*: Our study revealed significant clinical and radiological improvement. The CE angle improved from a mean value of 10° (range 2–17) pre-operatively to 32.785° (range 18°–40°) post-operatively. The AI improved from a mean value of 32° pre-operatively to a mean value of 13.89° post-operatively. HHS increased from a preoperative mean value of 74.80° to a post-operative mean value of 90.67°. Also, there was a significant improvement in ROM (abduction and internal rotation). LLD improved from a mean value of 2.60 cm preoperatively to a mean value of 0.37 cm postoperatively. Delayed union was found in 3 cases. No cases of osteonecrosis or neurovascular complication were encountered in our study. *Conclusion*: The modified TPO technique using dual incisions can be considered safe and effective, providing adequate coverage of the femoral head in acetabular dysplasia with less surgical time, satisfactory functional outcomes, and minimal complications.

Level of Evidence: IV

## Introduction

Acetabular dysplasia is a common disorder that is frequently researched and can be successfully treated. It presents as an isolated form or as a prerequisite for subluxation or complete hip dislocation (Developmental dysplasia of the hip). Insufficient femoral head coverage may develop even in cases of a well-developed acetabulum due to the changes in the femoral head (Legg-Calve-Perthes disease, avascular necrosis of femoral head) [1].

Management of acetabular dysplasia depends on the age of presentation. The best results can be gained if treatment is initiated at an early age, preferably before 4 years of age. The main aim of treatment is to attain stability of the femoral head inside the acetabulum at the earliest possible age with minimal complications [2].

Several types of acetabular osteotomies have been used for hip reconstruction in residual acetabular dysplasia in young adolescent patients including redirectional, reshaping, and salvage pelvic osteotomies. The aim of performing the procedure is to achieve adequate coverage of the femoral head and to increase congruence of the hip joint to preserve the hip range of motion and delay degenerative changes [3].

Triple pelvic osteotomy (TPO) includes osteotomies through the ilium and both pubic rami. It is indicated in children and adolescents with closing or closed triradiate cartilage. Although this osteotomy allows surgeons to redirect the acetabulum, the amount of correction remains questionable. This is mainly due to the strong muscular and ligamentous attachments around the pelvis in this age group, especially the sacrospinous and the sacrotuberous ligaments [3].

**Figure 1:**
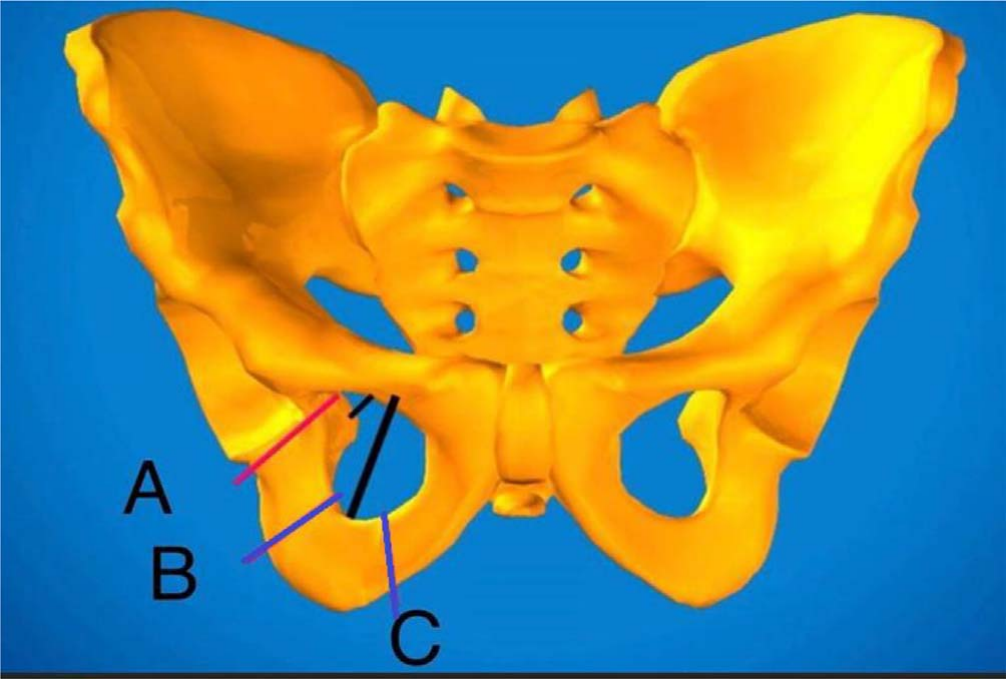
Sites of the ischial cuts. We used the infra-acetabular cut (red line-A), lateral to sacrospinous and sacrotuburous ligaments (black lines).

The main objective of this study is to highlight a surgical technique of TPO through double incisions with safety regarding nearby neurovascular structures. It evaluates the short-term clinical and radiologic outcomes in children and young adolescent patients with residual acetabular dysplasia to bony hip reconstruction using modified TPO. The modification includes two points. First, the skin incisions have been modified as described by Vladimirov [4, 5]. Second, the site of ischial bone osteotomy has been modified in accordance with the Li et al. [6] technique that fashions the far ischial cut lateral and proximal to the sacrospinous and sacrotuberous ligaments. This permits less tethering of the acetabular fragment by both ligaments enabling more free movement for better femoral head coverage (Figure 1).

## Material and method

This is a retrospective case series conducted on 15 hips in 15 patients presenting from 2018 to 2023 suffering from residual acetabular dysplasia and subjected to TPO. The mean age at the time of surgery was 11.85 years, SD = 4.67 (range 8°–23°). Cases were 10 males (66.7%) and 5 females (33.3%). Affected sides were 8 left (53.3%) and 7 right (46.7%). All patients were followed-up for 2 years with a mean of 36.533 months (SD = 8.82; range 24–60 months) (Table 1).

**Table 1:** Master sheet of 15 patients.

#	Age\years	Sex	Affected side	Complications	Preoperative			Postoperative											Follow-up
					Clinical			Radiological			Functional	Clinical			Radiological			Functional	
					Abduction range (Affected side/Normal side)	IR (Affected side/Normal side)	LLD (CM)	A.I	CE	FHEI, femoral head extrusion index	HHS	Abduction range (Affected side/Normal side)	IR (Affected side/Normal side)	LLD (CM)	A.I	CE	FHEI, femoral head extrusion index	HHS	
1	16	Female	Left	None	15°/30°	10°/40°	1.5	40°	9	75%	56	25°/30°	40°/40°	–	25	34	10%	84	26 m
2	8.3	Male	Right	None	20°/50°	50°/90°	–	35°	15	50%	88	50°/50°	70°/90°	–	15°	40	0%	97	28 m
3	23	Female	Right	Pubic osteotomy delayed union	15°/35°	15°/70°	2.8	35°	5	80%	53	25°/35°	35°/70°	0.5	10°	18	0%	77	34 m
4	8.9	Male	Left	None	10°/40°	15°/60°	1.2	30°	10	50%	89	40°/40°	45°/60°	–	5°	35	0%	97	40 m
5	8.2	Male	Right	None	30°/50°	20°/30°	–	20°	13	70%	85	50°/50°	30°/30°	–	8°	34	0%	95	30 m
6	9.2	Female	Right	Pubic osteotomy delayed union	35°/50°	20°/40°	–	30°	10	90%	80	50°/50°	20°/40°	–	15°	38	0%	90	36 m
7	11	Male	Left	None	30°/40°	30°/40°	1.5	25°	7	80%	76	40°/40°	40°/40°	–	10°	30	0%	89	44 m
8	8	Male	Left	Ischial osteotomy delayed union	10°/50°	15°/35°	1	30°	17	60%	90	40°/50°	25°/35°	–	10°	40	0%	98	38 m
9	20	Male	Left	None	15°/40°	40°/60°	3	40°	2	45%	63	40°/40°	55°/60°	0.3	20°	20	0%	89	42 m
10	10.3	Male	Right	None	20°/40°	20°/40°	1.2	50°	11	80%	72	37°/40°	40°/40°	–	25°	35	8%	85	60 m
11	8	Male	Left	None	20°/50°	50°/90°	2	35°	14	85%	69	45°/50°	80°/90°	–	15°	38	0%	93	36 m
12	13	Female	Right	None	15°/50°	15°/30°	1.9	25°	12	70%	67	40°/50°	35°/30°	–	5°	40	0%	93	39 m
13	10	Female	Right	None	20°/50°	30°/40°	1	20°	10	75%	76	50°/50°	35°/40°	–	10°	35	0%	90	31 m
14	9	Male	Left	None	15°/40°	40°/70°	0.5	40°	14	45%	88	40°/40°	60°/70°	–	25°		0%	96	24 m
15	14.9	Male	Left	None	25°/50°	0°/30°	2	25°	5	50%	70	50°/50°	25°/30°	0.3	10°	22	5%	87	40 m

**Figure 2:**
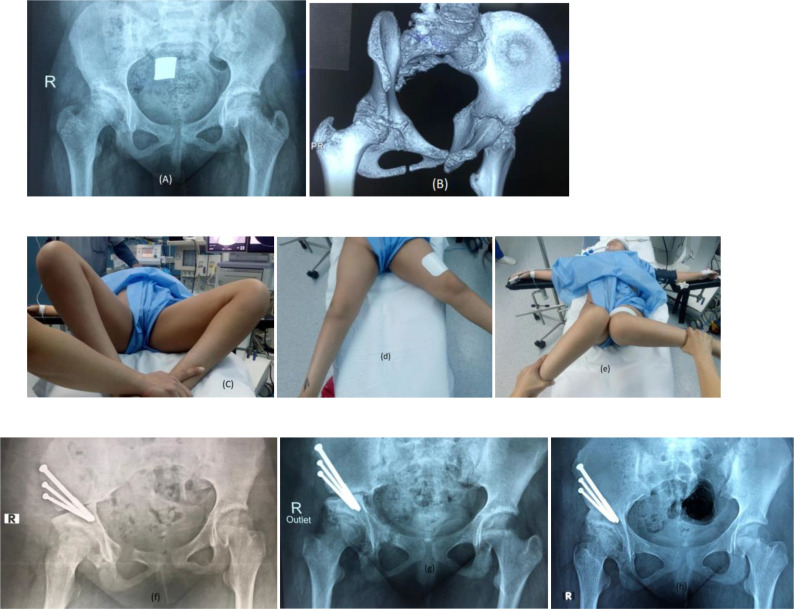
(a) Right hip dysplasia in 9.2-year-old-female. (b) 3D reconstruction demonstrates lateral acetabular wall deficiency. (c) Pre-operative clinical examination revealed limited hip abduction with knee flexion on rightt side. (d) Limited hip abduction with knee extension. (e) Limited hip internal rotation on right side. (f) Immediate post-operative pxr. (g) After 1 month. (h) After full union and remodeling.

The chief complaint noted by the patients on presentation was pain during activities of daily life. On physical examination, there was limping, limited hip internal rotation (IR), limited hip abduction, and limb length discrepancy (LLD). The mean abduction range in the affected side was mean 19.67 (SD = 7.43) preoperatively, compared to the unaffected normal side mean 44.33 (SD = 6.779). The mean IR in the affected side was 24.67, (SD = 14.82) preoperatively, compared to the normal side mean 51.00 (SD = 20.891) (Figures 2c–2e). Mean Harris Hip Score (HHS) is preoperatively 74.80 (range 53–90, SD = 11.977).

**Figure 3:**
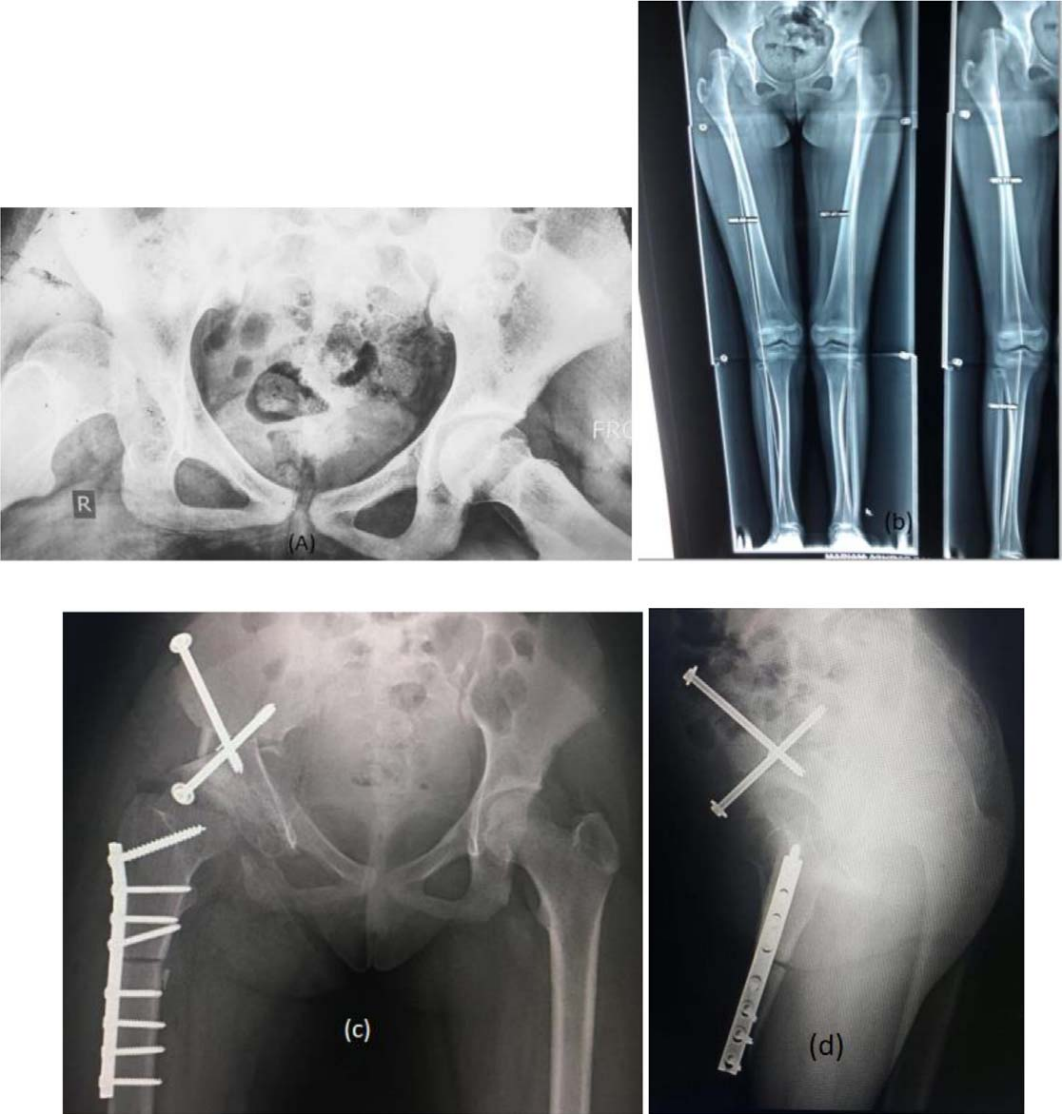
(a) PXR frog lateral demonstrates right hip dysplasia in 13-year-old female. (b) Standing long film demonstrates limb length discrepancy secondary to RT hip subluxation. (c, d). Immediate post-operative plain X-ray AP and lateral of TPO and femoral shortening.

Radiographs were done for all patients in the form of plain X-rays of the pelvis showing both hips from an antero-posterior view in neutral rotation, frog lateral, and abduction with internal rotation views. We recorded the acetabular index (AI), center-edge angle (CE), and femoral head extrusion index (FHEI). CT with three-dimensional (3D) analysis was done to assess acetabular dysplasia, acetabular wall deficiency and to exclude femoral head arthritis (exclusion criteria) (Figures 2a and 2b). Also, PXR of both lower limbs, from hip to ankle in a standing view was done to measure LLD (Figure 3b).

### Surgical technique

Triple pelvic osteotomy described by Tonnis [7] was done with two modifications. First is the Vladimirov modification [4, 5] that incorporates an accessible and reliable approach.

**Figure 4:**
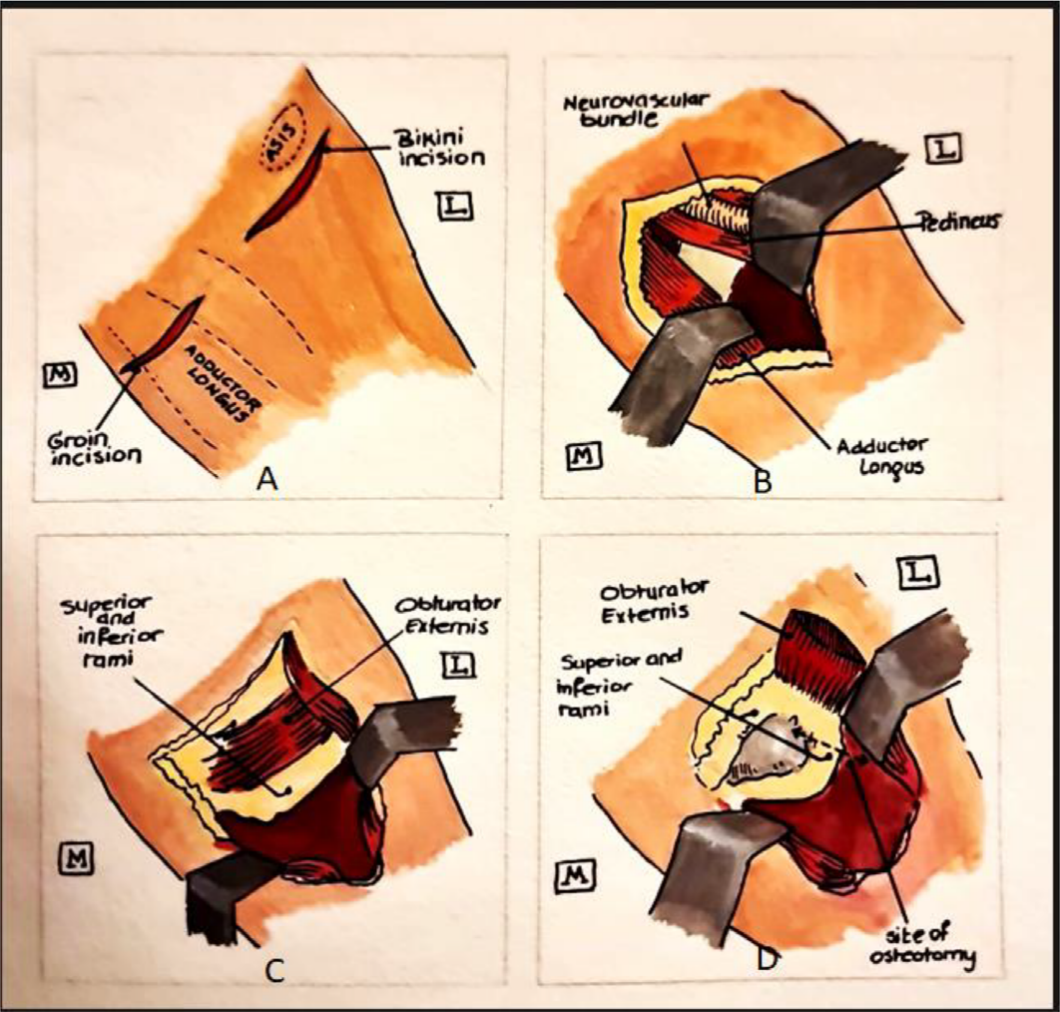
Drawing illustrating dissection relative to anatomy (a) medial and lateral skin incisions, (b) dissection between adductor longus and pectineous, (c) exposure of obturator externis and pubic rami, (d) elevation of obturator externis to expose rami and site of ischial cut represented by dotted line.

The patient is positioned supine and draped from subcostal to foot. First, the medial incision is made at the groin along a parallel line 0.5–1 cm distal to the groin skin crease and crossing over the adductor longus. Deep Dissection between the adductor longus and pectineus muscles is carried out and deep fascia is exposed. Next, the adductor longus muscle is retracted medially and the pectineus muscle is retracted laterally protecting the femoral neurovascular structures on the anterior aspect of the muscle. The obturator externus muscle is then elevated from its insertion on the pubic ramus and the superior pubic ramus is then sub-periosteally exposed (Figure 4).

The Osteotomy line of pubic cut was defined as the line between the anterior aspect of anterior obturator tubercle and the projection of the highest point of obturator sulcus on the superior pubic ramus. The starting point of the osteotomy was marked by an osteotome placed under an image intensifier. Performing the pubic osteotomy proceeded in a caudal to cranial direction from distal to proximal to avoid the corona mortis that is present in 30% of population.

Dissection was then directed and deepened laterally to the ischium. In the region between the ischium and pubis arms, the obturator externus was then removed subperiosteally and retracted anteriorly. Thus, the obturator vessels are protected by retracted muscle fibers. Just inferior to the acetabulum, the ischium was exposed subperiosteally.

On the body of ischium, the osteotomy line was identified as the line between the lower border of the posterior obturator tubercle and the highest point of the ischial spine.

An osteotome was advanced from lateral to medial after palpating the posterior obturator tubercle to avoid pudendal vessels and the sciatic nerve injury during the osteotomy exit, staying proximal to the scarotuberous and scarospinous ligaments as modified by Li et al. [6] (Figure 4d), but with no resection of a 1 cm segment of inferior ramus as in their procedure.

This allows free movement of the acetabular fragment for better and easier femoral head coverage. It also allows better bony union without affecting the acetabular movement.

The iliac bone is approached through the second Smith-Peterson’s incision (Bikini). The Anterior Inferior Iliac Spine (AIIS) is identified and the sciatic notch is protected inside and outside the pelvis. The iliac wing is osteotomized down to the sciatic notch by a power saw. The deep part of the osteotomy is completed with an osteotome. A Steinmann pin was inserted in the anterior inferior iliac spine to mobilize the fragment. The fragment is flexed to cover anteriorly. Lateral coverage is achieved by abducting the fragment and version is addressed through internal rotation of the fragment. Retroversion was avoided which is created by external rotation of the fragment.

Once the desired correction is achieved guided by the C-arm, the fragments are fixed by cannulated screws (4–7 mm) according to age and iliac bone size. Once fixation is stabilized, hip range of motion is checked to look for impingement or intra-articular penetration. Radiographs are taken in both AP and false profile view to check for the adequacy of correction obtained and to rule out retroversion.

A separate lateral thigh incision was used if concomitant femoral osteotomy was needed (femoral shortening osteotomy was required in three patients) in cases with broken shenton’s line in order to achieve containment, as we did not include incongruent or irreducible hip joints in our study (exclusion criteria).

Partial weight bearing using walking aids were allowed after pain subsidence postoperatively and continued for 6 to 8 weeks. Full weight bearing was then allowed without aids after bony union was achieved and assessed both clinically and radiologically.

## Results

The data presented by our study revealed significant clinical and radiological improvements. There is a statistically significant difference in mean HHS which increased from a preoperative mean value of 74.80 (range 53–90, SD = 11.977) to a post-operative mean value of 90.67 (range 77–97, SD = 5.82, p < 0.001) (Table 2).

**Table 2:** Comparison between preoperative and postoperative data.

Abduction range	Mean	*N*	SD	Standard error mean	P value	95% CI
Preoperative abduction range affected side	19.67	15	7.432	1.919	0.000	−26.01 to −17.59
Postoperative abduction range affected side	41.4667	15	8.24506	2.12886		
Preoperative IR affected side	24.67	15	14.816	3.826	0.000	−22.77 to −12.55
Post operative IR affected side	42.3333	15	17.09915	4.41498		
Preoperative LLD in cm	2.600	3	0.5292	0.3055	0.17	0.98 to 3.48
Post operative LLD in cm	.3667	3	0.11547	0.06667		
Preoperative AI	32.00	15	8.409	2.171	0.000	15.52 to 20.75
Post operative AI	13.8667	15	6.96795	1.79912		
Preoperative CE	10.00	14	4.188	1.119	<0.001	−25.22 to −20.34
Post operative CE	32.7857	14	7.51592	2.00871		
Preoperative FHEL	62.33	15	22.109	5.709	0.000	48.79 to 72.81
Post operative FHEL	1.5333	15	3.31375	0.85561		
Preoperative HHS	74.80	15	11.977	3.093	0.000	−20.07 to −11.66
Post operative HHS	90.6667	15	5.82687	1.50449		

Clinically, there was a statistically significant difference in the mean abduction range which increased in the affected side from a mean value of 19.67 (SD = 7.43) preoperatively to a mean value of 41.47 (SD = 8.24) postoperatively (*p* < 0.001). Also, the mean IR increased in the affected side from a mean value of 24.67 (SD = 14.82) preoperatively to a mean value of 42.33 (SD = 17.10) postoperatively (*p* < 0.001). There was a statistically significant difference in mean LLD that decreased from a mean value of 2.60 cm (SD = 5.31) preoperatively to a mean value of 0.37 cm (SD = 0.12) postoperatively (*p* < 0.001) (Table 2).

**Figure 5:**
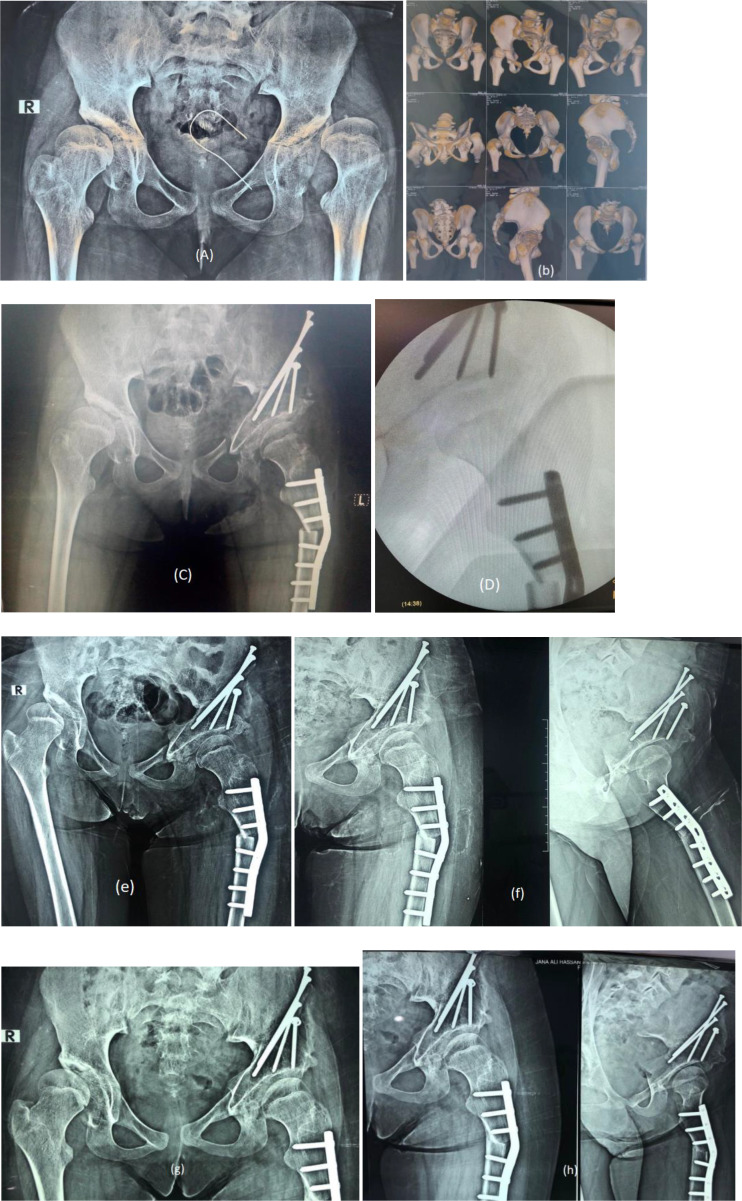
(a) Preoperative plain X-ray AP pelvis of 14 years old female with left hip dislocation and acetabular dysplasia, (b) preoperative CT scan, (c, d) immediate postoperative plain X-ray and intraoperative X-ray of femoral varus shortening osteotomy with TPO, (e, f) 1 month postoperative X-rays, (g, h) 6 months postoperative X-rays.

Radiologically, the CE angle significantly improved from a mean of 10° (range 2°–17°, SD = 4.19) pre-operatively to a mean of 32.785° (range 18°–40°, SD = 7.51, *p* < 0.001, *r* = 0.892) post-operatively. The AI was significantly improved from a mean of 32° (SD = 8.41) pre-operatively to a mean of 13.89° (SD = 6.97) post-operatively (*p* < 0.001, *r* = 0.828). There was a statistically significant difference in mean FHEL that decreased from a mean value of 62.33° (SD = 22.109) preoperatively to a mean value of 1.53° (SD = 3.13) postoperatively (*p* < 0.001) (Table 2 and Figures 2, 3, 5).

The mean Blood loss in our study was 395 Ml (range 240–700, SD = 141.81). The mean operative time was 73.67 min (range 40–110, SD = 23.10).

We had three cases (20%) with delayed union (a case with ischial osteotomy (6.7%) and two cases with pubic osteotomy (13.3%)). No cases of avascular necrosis of the femoral head or neurovascular complications were observed.

## Discussion

Acetabular dysplasia or inadequate acetabular coverage of the femoral head accounts for 20–40% of patients presenting with hip osteoarthritis (OA) [8]. This poor acetabular coverage of the femoral head is usually anterior and lateral which results in relative lateralization of the hip center of rotation and creates a mechanical problem in the form of increase contact stress between the femoral head and the dysplastic acetabulum. The goal of surgical treatment is to reduce load over the joint by increasing the contact area, relaxing the capsule and muscles about the hip to restore hip biomechanics, provide joint stability, improve function as well as gait, and reduce pain [8].

Our study aims to assess safety, efficacy, and short-term clinical outcome of the modified triple pelvic osteotomy (TPO) for correction of residual acetabular dysplasia using a dual approach. The data presented by our study revealed a significant clinical and radiological improvement, with improvement in HHS, radiological indices (CEA, AI), and postoperative hip ROM, with low rates of osteonecrosis and osteoarthritis.

The limitations of this study were the relatively small sample size and short follow-up duration. However, future research should be done with a larger sample size and a longer follow-up period to detect outcomes such as progression of OA and subsequent need for additional surgeries such as hip arthroscopy, repeat osteotomies, or conversion to THA. Also, CT scans can be used as an assessment tool for post-operative evaluation of the femoral head coverage, but it will be against ethical consideration to expose any of the patients to extra radiation.

Previous studies have demonstrated that early surgical correction of residual dysplasia with hip preservation surgeries will delay or prevent the onset of premature arthritis and delay the need for arthroplasty [3]. Grissom et al. [9] Al-Ghamdi et al. [10] described a triple pelvic osteotomy technique which allowed correction of inadequate acetabular coverage with very successful results as regards the clinical and radiological parameters. This technique was followed later on by some modifications in the approach by Vladimirov [11] which incorporates a more reliable and faster approach with no need to change the patient position and using the medial incision to achieve osteotomy of both rami [11].

In studies by van Hellemondt et al. [12], El-Tayeby [13], and Vukasinovic et al. [1] evaluating TPO in 138 hips, there was improvement in LCEA postoperatively to mean 22, also there was improvement in AI to mean 13. About 85% of patients had improved clinically (pain subsides) with some limitation in flexion, abduction, and internal rotation postoperatively during follow-up. In our study, the CE angle was improved from a mean of 10° (range 2°–17°, SD = 4.19) pre-operatively to a mean of 32.785° (range 18°–40°, SD = 7.51, *p* < 0.001) post-operatively. The AI was significantly improved from a mean of 32° (SD = 8.41) pre-operatively to a mean of 13.89° (SD = 6.97) post-operatively (*p* < 0.001, *r* = 0.828). This is more than the values portrayed by traditional triple pelvic osteotomy studies, suggesting that the modification of doing a far ischial cut lateral to the sacrospinous and sacrotuberous ligaments allows more free movement of the acetabular fragment for better and easier femoral head coverage.

Comparing our results with the periacetabular osteotomy (PAO), Sucato [14] reported the results of PAO in 24 adolescent hips, mean change of LCEA 27.5° (from 5.5 to 33), mean change of AI 18.5° (from 28.5 to 10), and HHS score had improved 1 year postoperatively by 10 points to reach 74.5. Also, most of the patients had functional improvement of the hip joint especially in abduction and flexion. Trousdale et al. [15] also reported the results of PAO done on 43 hips. The mean LCEA after surgery was 28°. The mean AI was 20°. The Harris Hip Score had improved by 24 points postoperatively, and in 10 years follow up 14% underwent THA, while the functional outcome improved in 90%. Asymptomatic heterotropic ossification was the most common complication that occurred in this study.

In Matta’s series of 58 hips, [16] the mean LCEA after surgery was 28°. Mean AI was 21.8°. During follow up, 13% underwent THA. About 77% had shown clinical improvement. The most common complication was pubic osteotomy nonunion in 17%. Bracken et al. [17] published a series on 123 hips. The mean LCEA after surgery was 23°. The mean AI was 17°. The HHS improved by 24 points postoperatively. During follow-up only 5% underwent conversion to THA and the most common complication was heterotropic ossification in 16%.

The comparable results between the two procedures regarding the radiological parameters indicates that the modified TPO technique using far ischial cut lateral to the sacrospinous and sacrotuberous ligaments (infra-acetabular), allows free movement of the acetabular fragment for better femoral head coverage. This technique is considered easier and faster requiring a lower learning curve than PAO, as the required osteotomies are more accessible and are done using only two incisions with no need for special equipment.

Studies on Shelf acetabuloplasty (Hirose et al. [18], Migaud et al. [19], Fawzy et al. [20], Bartoníček et al. [21], Dogan et al. [22], Ohashi et al. [23], Kotz et al., [24], and Vukašinović et al. [1]) were done on 180 hips. LCEA changed from 4.4° preoperatively to 44° postoperatively. The mean change of AI had reached 22°. There was clinical improvement in about 74% of cases, while during follow-up in 10 years postoperatively about 73 Patients (40.5%) had developed early OA and needed THA. So, we recommend reserving hip salvage procedures for cases with severely dysplastic and arthritic hips.

As periacetabular osteotomy is a difficult procedure, many modified interventions and approaches have been described to minimize complications. Ilioinguinal, modified Smith–Peterson approaches and direct anterior osteotomy may extend to the intra-articular region or may lead to posterior column fracture. Damage to blood vessels is also commonly seen [25]. However, the safety of this technique lies in advocating palpable anatomic landmarks (obturator sulcus, anterior, and posterior obturator tubercles) that can be approached through a medial approach keeping the nearby neurovascular bundle in a safe position during the approach and the osteotomy pathway with less risk of intra-articular fractures. Using two incisions instead of three does not necessitate an extra time-consuming step intra-operatively for repositioning of the patients. Another benefit is that it has a lower learning curve than PAO with less blood loss and operation time [25].

## Conclusion

The modified TPO technique can be utilized to effectively achieve adequate coverage of femoral head in acetabular dysplasia with less time consumption and minimal complications. It is a simple, safe, efficient, and reproducible technique, with very satisfactory radiological parameters and functional outcomes.

## Data Availability

The datasets generated during and/or analyzed during the current study are available from the corresponding author on reasonable request.
